# OBITUARY: Giorgio Gambale (1955-2015)

**DOI:** 10.1186/s13017-016-0106-1

**Published:** 2016-11-10

**Authors:** Vanni Agnoletti, Marco Barozzi, Luca Ansaloni, Francesco Cancellieri

**Affiliations:** 1 Anesthesia and Intensive Care Maurizio Bufalini Hospital – Trauma Center, Cesena, Italy; 2 Emergency Department, Maurizio Bufalini Hospital – Trauma Center, Cesena, Italy; 3 General Surgery Department, Papa Giovanni XXIII Hospital – Trauma Center, Bergamo, Italy; 4 Anesthesia and Intensive Care Maggiore Hospital – Trauma Center, Bologna, Italy

**Fig. 1:**
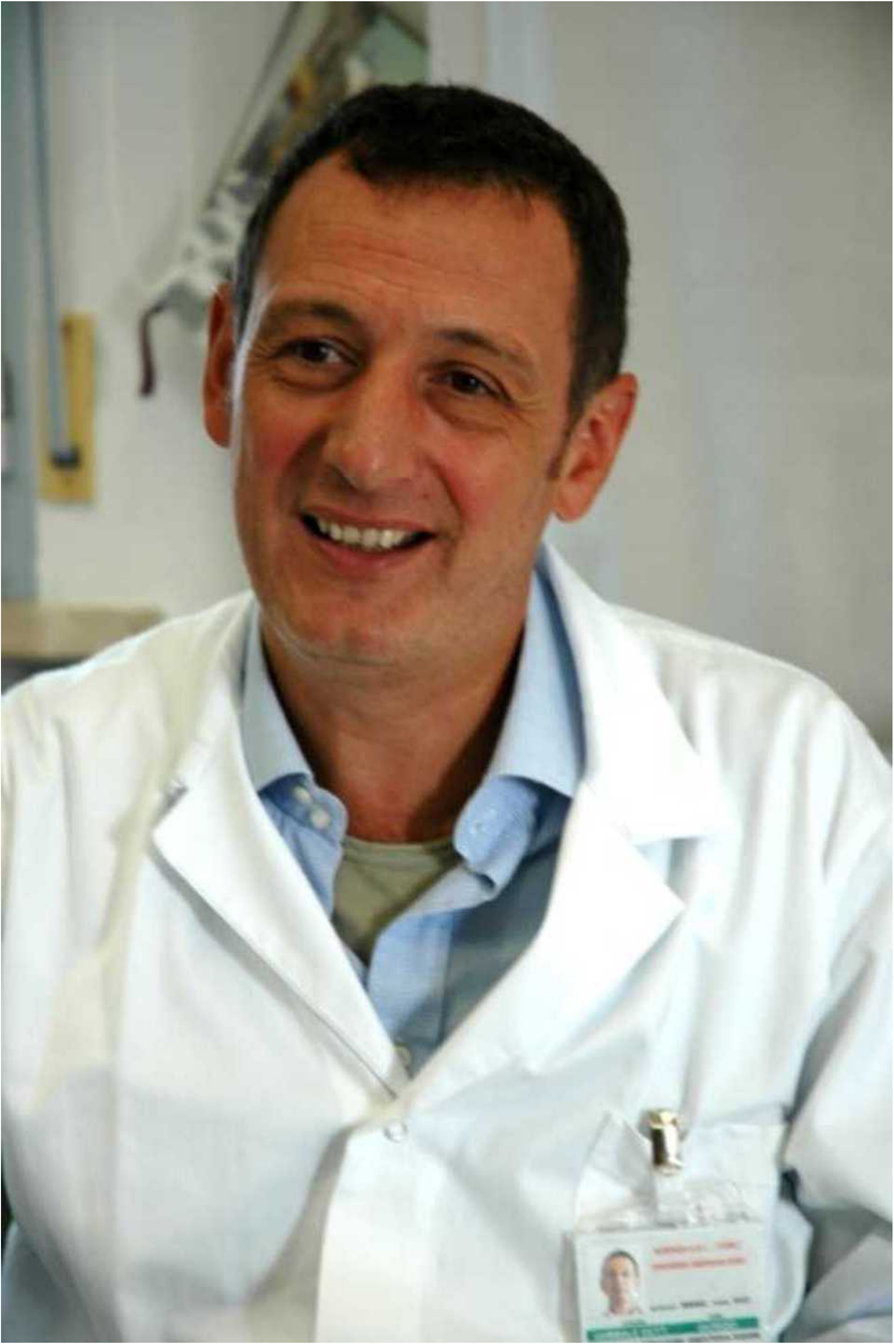
Giorgio Gambale (1955-2015)

Giorgio Gambale (Fig. 1) was born November 14, 1955 and died October 16, 2015 at his home in Bologna, Italy, surrounded by his family and friends after a one year battle with cancer. He received his medical degree in Bologna in 1980 and he got a postgraduate degree in Anesthesia and Intensive Care in 1983 and in Pneumology in 1987. He worked at Maggiore Hospital in Bologna where he gave a substantial contribution in setting up and running the Trauma Center. In 2005 he became the head of Anesthesia Department at Morgagni Pierantoni Hospital in Forlì (Italy) and in 2011 he was awarded by the European Institute of Public Administration for the project about the management of the surgical process. Afterwards in 2013 he moved to Cesena (Italy) to become head of Emergency and Trauma Department of Maurizio Bufalini Hospital.

He was the author of more than 50 peer-reviewed publications and in 2015 of a book titled “Medicine according to Captain Zantax” where he, after being diagnosed with glioblastoma, tells his professional life and sums up the meaning of his commitment in medicine. Captain Zantax is the nickname his colleagues gave him in Bologna because of the calm and kind confidence he displayed even in the most chaotic situation in the Emergency Department; his confidence, in turn, affected all the people around him positively. Like ranitidine, he could reduce the amount of ‘acid’ among people around him.

Giorgio had an emotional leadership style; he was able to inspire, to lead. He stayed in the background when everything was going fine and always in the foreground when something went wrong. As a visionary man he suffered when people around him were not able to see the big picture, to imagine the hypothetical future he was distinctly seeing and fighting for.

His enthusiasm for the field of trauma was stimulating and contagious. He never lost the curiosity for medicine and for life. Giorgio believed in meritocracy and in the strength of young generation of doctors. He thought the future was for “hungry and foolish” people, no matter the age.

As a good teacher he refused an academic career because he wanted to be in the field, to remain a doctor. He truly believed in the figure of a mentor, in the act of transferring experience and passion to young generations.

We all miss his smile that was like a big hug when you were down or like an injection of energy when you were tired or discouraged; it was impossible not to smile back to him.

He was a leader, he was the mentor he was looking for, he was the “Captain of the ship” he would have liked to sail after retirement. He has been the boss we all wanted to meet in life; he has been a brother and a master of life and profession.

